# Cloaca-Like Anomalies in the Male: A Report on Two Cases

**DOI:** 10.1055/s-0042-1750409

**Published:** 2022-07-27

**Authors:** Amr AbdelHamid AbouZeid, Shaimaa Abdelsattar Mohammad, Marco Rady Sos, Nader Nassef Guirguis, Heba A. Mahmoud, Manal El-Mahdy

**Affiliations:** 1 Department of Pediatric Surgery, Ain Shams University, Cairo, Egypt; 2 Department of Radiodiagnosis, Ain Shams University, Cairo, Egypt; 3 Department of Pathology, Ain Shams University Faculty of Medicine, Cairo, Egypt

**Keywords:** cloacal variant, anorectal malformation, penoscrotal transposition, UroRectal Septum, UroRectal septum malformation sequence

## Abstract

“Cloaca” is a term used to describe an anomaly in the female where a single orifice is located in the perineum draining both urogenital and gastrointestinal tracts. Few reports used the same term “cloaca” to describe the counterpart anomaly in the male. We present two “male” cases of anorectal anomalies associated with significant penile deformity (caudally displaced penis) that were managed during the period between January 2010 and September 2021. Characteristically, both cases had a single “central” perineal orifice. The latter was located anterior to the predestined site of the normal anus and just beneath a caudally positioned hypospadiac phallus. The caudal displacement of the penis was strikingly obvious by the presence of severe form of penoscrotal transposition. Both cases were associated with a perineal swelling (hamartoma) just beside the central perineal orifice. The urethra was very short (like that in the female), besides the single perineal orifice, which makes the presentation very similar to cloacal anomalies.

## Introduction

The incidence of anorectal anomalies is about one every 5,000 live births, with different phenotypic expression among both sexes. 1 The great diversity of the spectrum represents a real challenge for establishing a classification system that includes all variants in both males and females. 2 Most recently, the Krickenbeck classification has been proposed representing the evolution of previous successive classifications, so that pediatric surgeons all over the world would speak a common language. 3 Anorectal anomalies are categorized clinically into major and other rare groups. 3 However, occasionally, we may face some rare variants that do not fit into the already known groups of anorectal anomalies. 4 5 This report describes one of these variants with peculiar genital deformity associating anorectal anomalies in the male.

## Case Presentation

We present two “male” cases of anorectal anomalies associated with significant penile deformity (caudally displaced penis) that were managed during the period between January 2010 and September 2021.

### Case 1

**Fig. 1:**
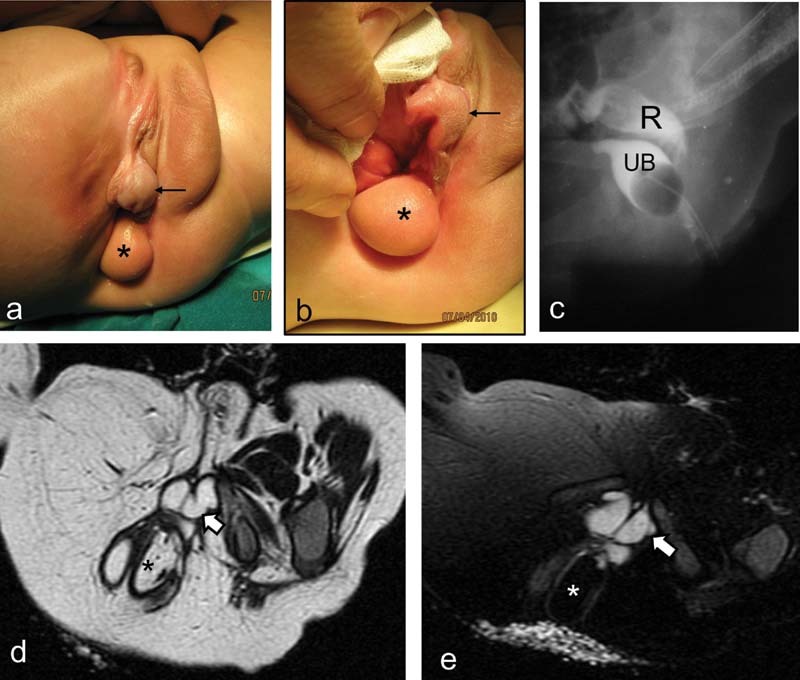
First case: ( **A, B** ) Clinical examination of the perineum (at the age of 7 months) demonstrates the caudally displaced hypospadiac phallus (black arrow), single “central” perineal orifice, and perineal lipoma (asterisk). Note: empty right scrotal compartment. ( **C** ) Contrast X-ray study: injection of contrast through distal colostomy demonstrating the rectum (R) that was not communicating with the urinary bladder (UB); the latter was opacified through separate contrast injection through perineal orifice. ( **D** ) Axial T2-weighted imaging (T2WI; pelvic magnetic resonance imaging [MRI]) demonstrating perineal lipoma (asterisk). Note the characteristic bending of the corpora cavernosa of the penis (thick white arrow), and the atrophy of the skeletal muscles of the hypoplastic right lower limb. ( **E** ) Axial T2WI (pelvic MRI) with fat suppression confirming the lipomatous nature of the perineal swelling (asterisk). Note: thick white arrow is pointing to corpora cavernosa of the penis.

The first case presented at birth with imperforate anus and intestinal obstruction for which a sigmoid colostomy was performed in the neonatal period. This case represented an extreme form of anorectal anomaly with severe sacral dysplasia, pelvic asymmetry, and deformed genitalia. Chromosomal analysis confirmed normal male karyotype (46 XY). Perineal examination revealed a caudally positioned hypospadiac phallus with severe penoscrotal transposition ( Fig. 1A and B ). A single wide perineal orifice proved to be the bladder neck directly communicating with the perineum without recognizable urethra in-between (vesico-perineal fistula). The urinary bladder was very small (underdeveloped) due to continuous leakage of urine through the wide and incompetent bladder neck. Distal colostogram demonstrated no communication with the urinary tract (imperforate anus without fistula) ( Fig. 1C ). A perineal swelling was located just beside the central perineal orifice which proved to be a lipoma on magnetic resonance imaging (MRI) ( Fig. 1D and E ). This case had other associated major anomalies: renal and testicular agenesis on the right side as well as poor lower limb development on the same side ( Fig. 1D ), asymmetrical lower vertebral dysplasia, and lipomyelomeningocele. Because of the severe distortion and asymmetry of pelvic anatomy, in addition to the presence of significant sacral dysplasia and spinal anomalies, a very poor prognosis for continence (both urinary and fecal) was expected in this case. After counseling with the parents, we decided to leave the patient with colostomy, while continent urinary diversion was performed later at the age of 4 years. The ureter of the single left kidney was disconnected from the hypoplastic bladder, and then was reimplanted in a pouch formed from intestine (ileocecal pouch), while the appendix was used as a conduit for self-catheterization (Mitrofanoff principle). The parents were instructed to keep on long-term follow-up at nephrology clinic for his special renal condition (single kidney draining into pouch of intestine). This patient is now 11 years old; and he is doing well at school. He is continent to urine through regular self-catheterization, and his single left kidney shows normal sonographic appearance at follow-up. He still has a colostomy, while the lipomyelomeningocele was managed conservatively. He underwent above knee amputation for the poorly developed right lower limb, and he is walking using artificial limb (right side) and crutches. He is now counseling for possibility of some sort of corrective surgery for the external genitalia.

### Case 2

**Fig. 2:**
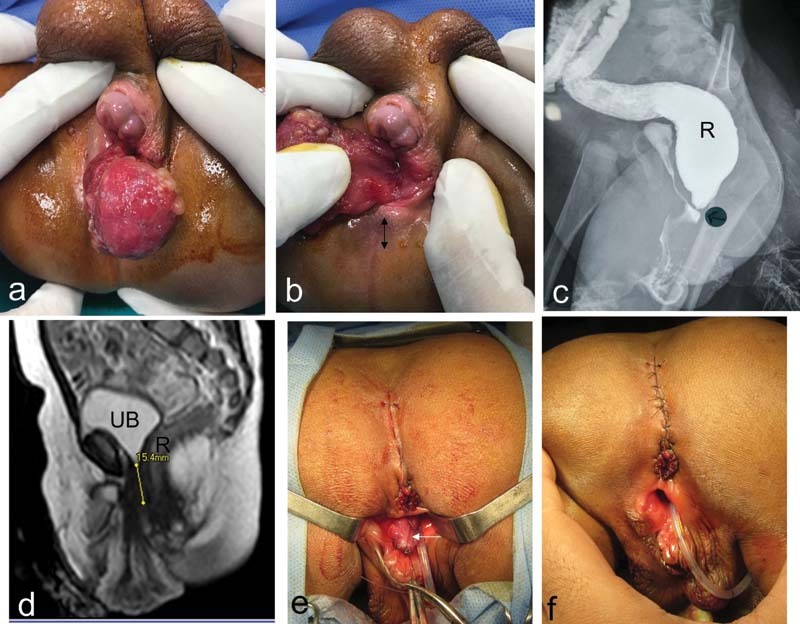
The second case: ( **A, B** ) Clinical examination of the perineum demonstrates the caudally displaced hypospadiac phallus and perineal swelling. Note: The presence of a single “central” perineal orifice anterior to the predestined site of the normal anus (double arrowhead line). ( **C** ) Contrast X-ray study: injection of contrast through distal colostomy demonstrating the rectum (R) communicating with the urethra to form a common perineal exit. ( **D** ) Mid sagittal T2-weighted imaging (pelvic magnetic resonance imaging) demonstrating the urinary bladder (UB) and rectum (R). Note the funneling and descent of the bladder neck in the pelvis down to the level of the lower end of pubic symphysis; also, the measured distance at which the rectum joins with the urethra is approximately 15 mm below the bladder neck. ( **E, F** ) Operative photos for posterior sagittal anorectoplasty and excision of perineal swelling (white arrow is pointing to stump after excision of perineal swelling).

**Fig. 3:**
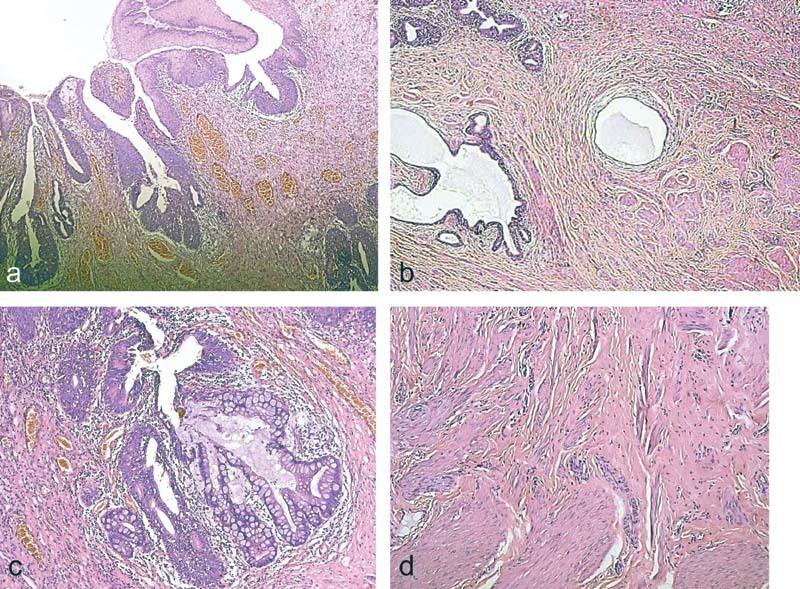
Histopathological examination of the excised perineal swelling (case 2): ( **A** ) Polypoid lesion covered by keratinized stratified squamous epithelium with many anal ducts lined by transitional epithelium which are opening on the surface. The core contains multicystic spaces lined by different types of epithelium ( **B** ), mucin secreting glands, ( **C** ) with disorganized bundles of smooth muscles in between ( **D** ). Hematoxylin and eosin; original magnification: a and b (×40), c (×100) and d (×200).

The second case was referred to our hospital at the age of 9 months with a sigmoid colostomy performed elsewhere at birth. Perineal examination revealed similar anatomical derangement ( Fig. 2 ): single perineal orifice, caudally positioned hypospadiac phallus, severe penoscrotal transposition, in addition to a perineal swelling covered with mucous membrane. Chromosomal analysis confirmed normal male karyotype (46 XY). Imaging studies demonstrated the rectum joining with the urethra 1.5 cm below the bladder neck to form a common excretory orifice just distal to their confluence. Compared with the first case, this case represented a milder form of the anomaly, not only for the absence of major associated anomalies but also for the presence of a recognizable segment of proximal urethra below the bladder neck. However, the urethral segment was too short (like the female urethra); and the bladder neck was still wide and was located at a lower level in the pelvis opposite the distal end of the pubic symphysis ( Fig. 2D ). At the age of 18 months, posterior sagittal anorectoplasty was performed to mobilize the rectum and reposition it backward within the sphincter muscle complex, in addition to excision of the perineal swelling ( Fig. 2E , F ). Histopathological examination of excised perineal swelling was compatible with hamartomatous polyp ( Fig. 3 ). Correction of the penile deformity will require staged reconstruction.

## Discussion

This report is about two peculiar cases, which we have encountered during management of many boys with “usual” forms of anorectal anomalies over the last 10 years. Characteristically, both cases had a single “central” perineal orifice. The latter was located anterior to the predestined site of the normal anus and just beneath a caudally displaced hypospadiac phallus. The caudal displacement of the penis was strikingly obvious by the presence of severe form of penoscrotal transposition. Both cases were associated with a perineal swelling (hamartoma) just beside the central perineal orifice. Through studying these two cases, as well as reports on similar cases in the literature, 4 5 6 7 8 9 we have tried to define this peculiar condition and clarify mixing and confusing terminology for such rare situation.

The cloacal membrane represents a key structure whose fate has been a matter of debate among researchers. 10 Developmental defects at the caudal side of the cloacal membrane result in usual forms of anorectal anomalies. On the other hand, defects at the cranial side are responsible for urethral anomalies described in the posterior cloaca /absent penis spectrum. 11 In this report, a dual developmental defect at both sides of the cloacal membrane can provide explanation for this peculiar clinical presentation that combines both anorectal and urethral defects in the same subject. The cranial end of the cloacal membrane meets with the genital tubercle forming a fixed point; the latter has been described to never lose contact with the tip of the developing phallus. 10 In our case, failure of formation of the distal (anterior) urethra reflects poor development of the cranial part of the cloacal membrane that remained anchoring the tip of the phallus to the perineum. This would provide explanation for the characteristic penile deformity that we have found to be present in these cases without other evidence of defective virilization.

Reviewing the literature, few reports used the term “cloaca” to describe similar cases in the male 4 6 ; meanwhile, other reports used alternative nomenclature: “UroRectal Septum Malformation Sequence 7 ,” and “Exstrophy of the urorectal septum 8 .” The term “Cloaca” is used almost exclusively in the female to describe a condition where the intestinal, genital, and urinary tracts join to form a common channel, which empties through a single perineal orifice. 12 In their report, Sharma and Gupta identified cardinal features to justify their unusual diagnosis of cloaca in the male that included passage of meconium stained urine through a single perineal orifice. 4 We do agree that the term “UroRectal Septum Malformation Sequence” is so generalized 13 14 ; however, the term “cloaca” is still unfamiliar and confusing to be used in the male. Failure of development of the anterior urethra is what makes this anomaly peculiar from other anorectal anomalies in the male. The urethra becomes very short (like that in the female), besides the single perineal orifice, which makes the presentation very similar to cloacal anomalies. Therefore, we may rather call it a “cloaca-like” anomaly in the male.

Away from conflicts about semantics, we should have a clear identification of the different components of this complex anomaly to avoid confusion especially when dealing with such cases for the first time. The anorectal component of the anomaly should be managed in the same way according to its type. Fecal diversion (colostomy) is usually performed at birth as a preliminary step. Contrast X-ray studies (distal colostogram) and endoscopy through common perineal orifice are essential to delineate the communication between the gastrointestinal termination and the genitourinary tract. MRI can be very useful to uncover the internal pelvic anatomy. The lower urinary tract may be significantly distorted requiring staged and complex reconstructive procedures to achieve social continence. The overall prognosis will depend on the extent of associated anomalies (skeletal/vertebral and spinal).

## Notes

Importance for a pediatric surgeon We present a rare anorectal anomaly in boys associated with significant genital deformity and bizarre perineum that may cause confusion on presentation. A detailed anatomical and embryological analysis is provided based on cross-sectional imaging (MRI) studies of two cases. The operative and histo-pathological findings are also provided.
